# Slow wave electroencephalogram spectral properties during adaptation of a new light dark cycle in cynomolgus monkeys

**DOI:** 10.1093/sleepadvances/zpag020

**Published:** 2026-02-10

**Authors:** Ruitong Jiang, Julian Low, Martha H Vitaterna, Karrie Fitzpatrick, Douglas J Weber, Darcy M Griffin

**Affiliations:** Neuroscience Institute, Carnegie Mellon University, Pittsburgh, PA, United States; Center for Neural Basis of Cognition, Pittsburgh, PA, United States; NeuroMechatronics Lab, Carnegie Mellon University, Pittsburgh, PA, United States; NeuroMechatronics Lab, Carnegie Mellon University, Pittsburgh, PA, United States; Department of Mechanical Engineering, Carnegie Mellon University, Pittsburgh, PA, United States; Center for Sleep and Circadian Biology, Northwestern University, Evanston, IL, United States; Department of Psychiatry & Behavioral Sciences, University of Minnesota, Minneapolis, MN, United States; Neuroscience Institute, Carnegie Mellon University, Pittsburgh, PA, United States; Center for Neural Basis of Cognition, Pittsburgh, PA, United States; NeuroMechatronics Lab, Carnegie Mellon University, Pittsburgh, PA, United States; Department of Mechanical Engineering, Carnegie Mellon University, Pittsburgh, PA, United States; Neuroscience Institute, Carnegie Mellon University, Pittsburgh, PA, United States; Center for Neural Basis of Cognition, Pittsburgh, PA, United States; NeuroMechatronics Lab, Carnegie Mellon University, Pittsburgh, PA, United States

**Keywords:** circadian rhythm, light dark cycle shifts, electroencephalogram, entrainment, wavelet coherence, delta power

## Abstract

**Study Objectives:**

Shifts in the light–dark cycle (L:D cycle) often trigger phase shifts in physiological data related to the sleep–wake cycle. Slow wave activity (delta) indicates sleep pressure and intensity. This study examines how delta power adapts to shifts in L:D cycle and the temporal dynamics of its coupling with rest-activity rhythms during re-entrainment.

**Methods:**

We collected electroencephalogram (EEG) and accelerometer data from three non-human primates during baseline and shifted (8-h delayed light-on) conditions. We derived delta power (0.5 ~ 4 Hz) using Fast Fourier Transform. To quantify changes in delta power dynamics following L:D cycle shifts, we calculated diurnal differences in delta power, per cent variance explained by time-of-day, circadian coupling with physical activity, and delta power activity transitions timing.

**Results:**

In both conditions, delta power exhibited a robust 24-h periodicity, and a significant portion of the variance (57.61% ± 6.99%) could be explained by time of day. We found an early transition of delta power in the first 2 days of the shifted condition, followed by realignment to the light-off time within 3 days after the shift. We used coherence analysis to reveal strong coupling between delta power and locomotor activity, with a consistent anti-phase relationship across baseline and phase shifted conditions.

**Conclusions:**

Our findings demonstrate that delta power adapts rapidly to environmental phase shifts while maintaining circadian rhythmicity and stable coordination with rest-activity rhythms. Here, we provide new insight into how neural and behavioral states remain aligned during circadian disruptions in a diurnal species.

Statement of Significance We studied how delta power, a slow-wave electroencephalogram (EEG) marker of sleep pressure, adapts to an abrupt 8-h shift in the light–dark cycle (L:D cycle) in cynomolgus monkeys. By continuously recording EEG and actigraphy, we found that delta power exhibited a stable 24-h rhythm, re-entrained rapidly to the new light–dark schedule, and maintained a consistent anti-phase relationship with physical activity. Wavelet coherence analysis revealed strong and persistent coupling between delta power and activity across both baseline and shifted conditions. These findings suggest that delta power reliably tracks rest-phase timing and remains tightly coordinated with physical activity patterns even during L:D cycle disruption in a diurnal animal model.

## Introduction

The circadian clock is an endogenous and autonomous pacemaker that regulates the timing of sleep and wakefulness. It is strongly influenced by external L:D cycles known as zeitgebers [1, 2]. In mammals, light signals the suprachiasmatic nucleus to regulate physiological processes such as hormone production, cardiovascular function, core body temperature, and mood, aligning them with a 24-h cycle [3–9]. The ensemble of these synchronized physiological processes forms the circadian system, with each process exhibiting its own circadian rhythm. Alterations to the L:D cycle shifts our exposure time to light or darkness; disrupting these homeostatic processes. This disruption leads to alterations in our sleep structure. Symptoms of these alterations include daytime sleep or insomnia, digestive issues, and affective disorder [10–13].

Non-human primates (NHPs) are a valuable translational model to study circadian system and sleep pattern disruptions from shifting L:D cycles. This is due to the fact that NHPs and humans share common genetics, brain circuitry, physiology, and diurnal sleep patterns [14]. In fact, the NHP model has been frequently used to investigate how factors such as drug interventions and genetic mutations influence sleep, providing insights that are difficult to obtain from human studies [15–18].

Electroencephalogram (EEG) is an essential tool for studying the dynamics of brain activity during sleep and wakefulness, as EEGs show continuous fluctuations that are correlated with different phases of circadian rhythms [19–21]. When studying the pattern of sleep stages with telemetry EEG, multiple studies have provided strong supporting evidence that NHPs and humans have similar compositions of sleep stages [22–25]. EEG can be divided into different bands of activity that reflect synchronous firing of populations of neurons. The slow-wave EEG activity (1 ~ 4 Hz), also known as delta waves, is widely recognized as the primary electrophysiological indicator of homeostatic sleep pressure [26, 27]. Quantitatively, delta power reflects the intensity of neural synchronization that correlates with recovery processes, making it a robust and continuous biomarker for sleep adaptation [26, 28]. Delta band amplitude increases as subjects stay awake for longer and dissipates as sleep cycles through non-rapid eye movement and rapid eye movement stages [28–31]. However, due to the restrained nature of traditional EEG recording methods, research in humans has primarily focused on delta band activity during discrete recording sessions. The alteration of continuous dynamics of delta band activity due to zeitgeber changes remains largely unexplored.

While delta power is recognized as a marker of sleep homeostasis, its temporal relationship to other circadian markers (such as physical activity) has not been well characterized under continuous, unconstrained conditions. Delta power is typically studied during consolidated sleep episodes, whereas physical activity can be continuously tracked and is commonly used to assess circadian phase [32–34]. Whether delta power remains tightly linked to rest-activity cycles across multiple days, particularly when the external L:D cycle shifts, remains unknown. Investigating this relationship under more naturalistic conditions can help determine whether delta power reflects an intrinsic process that remains coordinated with established circadian markers, even when environmental timing cues shift.

In the present study, we used wireless telemetry implants to record EEG and physical activity from freely behaving cynomolgus monkeys. We tracked delta wave adaptation during an 8 h phase-lagged L:D cycle designed to simulate acute jet lag. We recorded data for a continuous 12 days under normal L:D cycle conditions (baseline condition), and an additional 2 days before, and 8 days after an L:D cycle shift (shift condition). After the L:D cycle shift, we observed an early increase of delta power. As the animals adapted to the new L:D cycle, the timing of delta power increase progressively realigned with the light-off time. In addition to the temporal shifts, we observed that delta power remained strongly coupled with physical activity in an anti-phase relationship across both baseline and shift conditions. Here, we highlight the neurophysiological impact of acute jet lag conditions on EEG delta power, characterized by an early increase in delta power, and preserved temporal coordination with the rest-activity cycle.

## Materials and Methods

### *Subjects* 

We conducted all experimental procedures according to National Institutes of Health and the US Department of Health and Human Services guidelines as reported in the Association for Assessment and Accreditation of Laboratory Animal Care and the Guide for the Care and Use of Laboratory Animals. All procedures were approved by the institutional animal care and use committees at Carnegie Mellon University. We observed EEG delta power and physical activity in three adult male cynomolgus monkeys (*Macaca fascicularis*; >8 years old, weight range: ~8.2–8.9 kg). The animals were housed in a stable environment (temperature 21.5 ± 0.7°C, humidity 47%–55%) with a L:D cycle of 13 h of light and 11 h of dark (light on time 06:00 to 19:00). To achieve the L:D shift, we extended the light cycle by 8 h. Throughout all data recording conditions, the animals were fed commercial monkey chow (18 biscuits daily; LabDiet 5038) at ~06:00, 12:00, and 15:00 during baseline conditions and at ~14:00, 18:00, and 23:00 during shifted conditions. This feeding schedule is equivalent to the first hour, fifth–sixth hour, and eighth–ninth hour relative to light on (See the green vertical line markers in Figure 2, A, Figure 3, A). When the L:D cycle was extended, we provided three additional biscuits at 0:00. Fresh fruit and other novel food enrichment was provided daily at non-fixed times during the light period. Water was provided during and after task training (See the orange vertical line markers in Figure 2, A and B, Figure 3, A and B). Additional environmental enrichment was provided via chew toys, mirrors, videotapes, and music.

### *Surgery* 

We performed all surgeries in an aseptic environment with the animal under deep general anesthesia (1.5%–3% Isoflurane). Postoperatively, monkeys were given analgesics and antibiotics. The telemetry device (PhysioTel Digital L04 implant; Data Sciences International, St.Paul, MN, United States) consists of an electronics package sealed hermetically in a titanium enclosure, four pairs of lead wires corresponding to four different bipolar biopotential channels, a temperature sensor, and a three-axis accelerometer for actigraphy. We designated the biopotential channels to record electrocardiogram (ECG), electromyogram (EMG), and EEG from central and parietal/frontal scalp regions. We placed the electronics package in a pocket between the external and internal abdominal oblique muscle and secured it with sutures. The ground reference of these biopotential channels is located inside the implant body.

We tunneled the ECG leads subcutaneously from the abdominal pocket. We then positioned the positive lead near the lateral xiphoid process and the negative lead in the right pectoral region. For the EMG channel, we tunneled the leads subcutaneously from the abdominal pocket and placed them into the cranial aspect of the trapezius muscle. To prevent signal shorting, we separated the positive and negative leads by ~4 mm within the same muscle fiber group. We placed EEG channels on the central and frontal scalp region of two subjects (subject V and G) and the central and parietal scalp region of another subject (subject BF). For all EEG channels, we tunneled the EEG leads from the abdominal area to the skull using a trocar and cannula. We drilled two burr holes for each bipolar EEG channel according to the international 10-20 system. We placed a medical-grade stainless steel screw (Protech International, Inc.) in each burr hole. We secured the leads by wrapping the exposed metal ends around the base of the screws. We then secured each screw into the respective burr hole with dental acrylic. For the central scalp region, we placed the negative lead at the burr hole at Cz and the positive lead at the burr hole at C3 (subject BF) or C4 (subject V and subject G). For the frontal scalp region, we placed the negative lead at the burr hole at F3 and the positive lead at the burr hole at F4. For the parietal scalp region, we placed the negative lead at the burr hole at Pz and the positive lead at the burr hole at P3. To ensure adequate recovery, we initiated data collection 1 month after the telemetry implant surgery. For the purpose of this study, we only used the EEG data from the central scalp region.

### *Data recording* 

**Figure 1:**
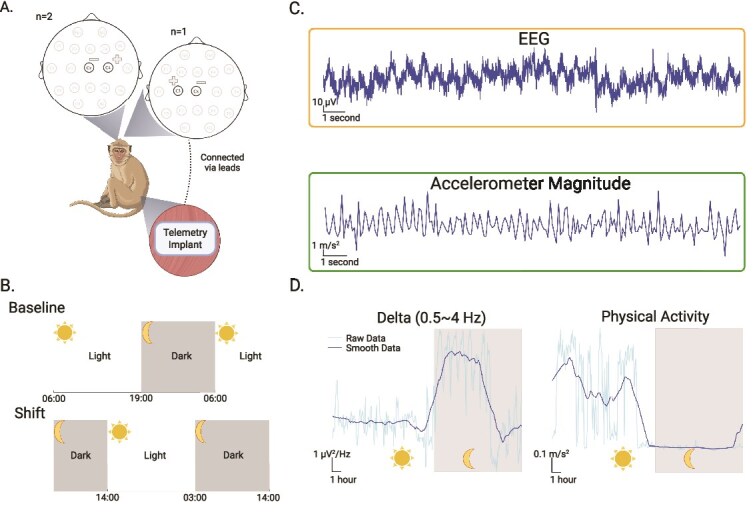
Experimental Schematic. (A) Telemetry device placement. The telemetry device was placed on the left side of the abdomen. EEG leads were tunneled from the abdominal area to the skull. In two animals, the positive lead was placed on C4, while negative lead was placed on Cz. In one other animal, the positive lead was placed on C3, while negative lead was placed on Cz. (B) Day-night cycle schematic on a 24-h timeline. In the baseline condition, the light turned on at 6:00 and turned off at 19:00. In the shifted condition, the light-on and light-off time was delayed by 8 h. Under this condition, the light turned on at 14:00, and turned off at 3:00. The ratio of light period and dark period was kept consistent at 13:11 in both conditions. In this study, one day is defined as the period of time from light on to the end of light off. (C) A 20 s sample of telemetry and accelerometer data from one animal (Monkey V). (D) One day time series of delta power (derived from EEG data) and physical activity. The light blue traces indicate the raw time series data. The dark blue traces indicate the smoothed data using a linear filter with a 36-samples moving window (3 h).

We recorded EEG signals and actigraphy continuously for 12 days in the baseline condition and an additional 10 days (2 days pre-shift and 8 days post-shift) in the shift condition. The telemetry implant sent the data wirelessly to a data transceiver (TRX-1; Data Sciences International) mounted to the top of each animal’s cage. We recorded and stored the data on a computer, through the data-exchange matrix (Physiotel Digital communications link controller; Data Sciences International) connected to each transceiver. The accelerometer provided acceleration data along the *x, y*, and *z* axes. We sampled EEG at 500 Hz, and actigraphy data at 10 Hz (Figure 1, C).

In the baseline condition, we programmed the lights to turn on at 6:00 and off at 19:00. In the shift condition, we programmed the lights to turn on at 6:00 and off at 19:00 for the first 2 days. On the third day, we programmed the lights to stay on through 19:00 and turn off at 3:00 the following day (day 4). After that, the program turned the lights on at 14:00 and off at 3:00 (Figure 1, B). We were unable to record the baseline and shift condition data sequentially due to biweekly cage and home room sanitization requirements. For cage sanitization, we temporarily removed the animals from their home cages, and dismantled all the attached recording equipment. After sanitizing, returning the animals to their home cages, and replacing the recording equipment, we allowed them a 2–3 h acclimation period before resuming data collection.

### *Data analysis* 

To calculate the daily rhythm of log spectral power, we segmented the EEG data into 10-s epochs with 50 per cent overlap. We removed data epochs that contained large sections (>1 s) of data dropout or clipping due to large artifacts in the physical activity (z-score > 4). We performed the Fast-Fourier transform on each epoch to generate the power spectral density of the signals in the epoch. We calculated the absolute power for the delta band (0.5 ~ 4 Hz). We selected the 0.5 ~ 4 Hz range as it is consistent with the definitions of the delta band in recent NHPs studies with telemetry recording [25, 35, 36]. We then averaged absolute power across a 5-min window to generate the time series data for the log spectral power of the delta band (Figure 1, D, left). We then conducted a two-sample *t*-test to compare the delta power between light and dark periods for both baseline and shift conditions. To characterize the dominant periodicities in the time series, we computed the power spectral density for each subject. We then divided each spectrogram by its total power for normalization. The spectra were converted to the period domain (1/f) to facilitate interpretation.

To calculate the physical activity, we replaced any data dropout points with the nearest non-missing values. We derived the actigraphy magnitude by taking the square root of the sum of squares of the acceleration data from the *x, y*, and *z* axes. To remove potential drifting issues in actigraphy data, we applied a fourth-order high-pass Butterworth filter with a cutoff frequency of 1 Hz. We then averaged the filtered actigraphy magnitude over a 5-min window (Figure 1, D, right).

We calculated the proportion of log spectral power variance explained by time of day using the Circa Diem toolbox [37]. In this method, the time series data are divided into multiple 30-min intervals, and the average of each interval ${y}_i$ is used as the center value of the interval. The value between each center point of the interval is calculated using linear interpolation, and the difference between the fitted value and observed values $y$ is ${y}_{\mathrm{residual}}$. We used the following formula to calculate the variance explained by the time of the day:


$$\mathrm{Variance}\ \mathrm{Explained}=\frac{\mathrm{Var}(y)-\mathrm{Var}\left({y}_{\mathrm{residual}}\right)}{\mathrm{Var}(y)}$$

We calculated the proportion of log spectral power variance for both baseline and shift conditions. In order to estimate the dominated period in daily log spectral power fluctuations, we conducted Fast Fourier Transform on the log spectral power time series data for both baseline and shift conditions.

We assessed the temporal relationship between EEG delta power and physical activity rhythms during both baseline and post L:D cycle shift by computing the wavelet coherence and phase angle using MATLAB’s *wcoherence* function. This function applies a continuous wavelet transform to both input signals and quantifies their localized time-frequency coherence, along with the relative phase difference at each frequency. We first z-scored normalized EEG delta power and physical activity signals. We applied zero-padding to both ends of each time series to ensure the entire data duration remained within the cone of influence of the wavelet transform. We then extracted coherence and phase angle values specifically within the 24-h circadian band. To focus on the strength of temporal offset regardless of lead/lag direction, we took the absolute value of the phase angle. We averaged both coherence and absolute phase values in 24-h bins to visualize daily dynamics across the recording period in both conditions.

We used the standardized and filtered data to define the Delta power transition point relative to the light-off time for each 24 h period. The Delta power transition point is associated with a transition from a state of wakefulness to a state of rest. We set the threshold for recognizing the Delta power transition point to zero in the z-scored delta power data. From this analysis, we set the activity transition for delta power as the first occurrence of the value falling below the threshold relative to the light-off time. We categorized Delta power transition points greater than 1 as “early,” those less than −1 as “late,” and those between −1 and 1 as “on-time.” To compare the distribution of Delta power transition point categories between baseline and shift conditions, we performed a chi-square test of independence. To establish baseline intervals for the Delta power transition points, we used the 5th and 95th percentile range of the baseline data. This interval allows for a robust comparison of daily deviations while accounting for natural variability within the baseline period.

## Results

### *Delta power dynamics in baseline condition* 

**Figure 2:**
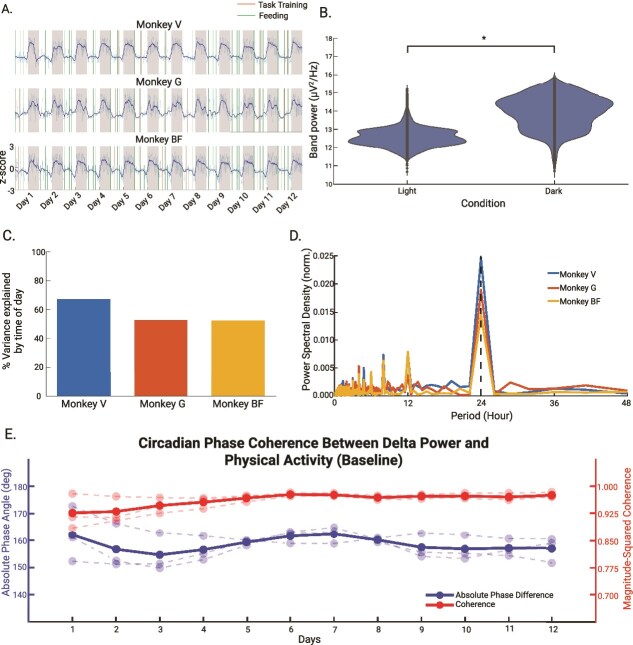
Diurnal patterns of delta power in the baseline condition. (A) Time series of delta power in the baseline condition (12 days, *n* = 3). Green and orange lines indicating feeding and task training time, respectively. Animals received their water during and right after the task training. We standardized delta power by taking the z-score. (B) The distribution of delta power across light and dark conditions. (C) The percentage of variance in delta power as explained by time of day for each animal. (D) Periodograms of delta power time series. We normalized each trace in the plot by the sum of the power across all periods. The dashed vertical line marks the peak power period (24 h) for each monkey. (E) Daily average of magnitude-squared coherence and absolute phase angle between delta power and physician activity in baseline condition (12 days). We computed wavelet coherence between the two signals, extracted values from the 24-h circadian band, and averaged coherence and phase angle for each 24-h bin. Solid lines represent the group mean (*n* = 3); dashed lines show individual animals.

During the baseline condition, we found a consistent daily fluctuation in delta power, synchronized with the light–dark cycle (Figure 2, A), such that delta band power was significantly higher in the dark period as compared to the light period (*t*(10325) = 97.81, *p* < .001; Figure 2, B). Thus, we observed the expected diurnal pattern in delta band power and the physical activity of all three NHPs. Time of day fits explained a significant proportion of variance in delta power (*p* < .001, temporal shuffling test, Figure 2, C), and the dominant periodicity of the delta power fluctuation indicates a robust 24-h period cycle (Figure 2, D). We next examined the phase relationship between delta power and physical activity. Using wavelet coherence analysis, we observed consistently high coherence values between the 2 signals across all 12 baseline days (group coherence = 0.96 ± 0.02; individual: Monkey V = 0.96 ± 0.02, Monkey G = 0.98 ± 0.01, Monkey BF = 0.95 ± 0.03; Figure 2, E). The absolute phase difference between delta power and physical activity remained close to 180 degrees throughout the baseline period, indicating a stable anti-phase relationship (group absolute phase angle = 158.42 ± 2.51; individual: Monkey V = 162.25 ± 3.84, Monkey G = 155.93 ± 4.27, Monkey BF = 157.08 ± 4.72). These high coherence values confirm the expected robust and consistent circadian coupling between EEG-derived delta power and physical activity during the baseline period. Additionally, we observed a stable anti-phase relationship between these signals, indicating that delta power is high during periods of low physical activity and low during periods of high physical activity. Our findings substantiate that delta power has a consistent coupling with rest and sleep-related processes in unperturbed conditions.

### *Delta power dynamics after the L:D cycle shift* 

**Figure 3:**
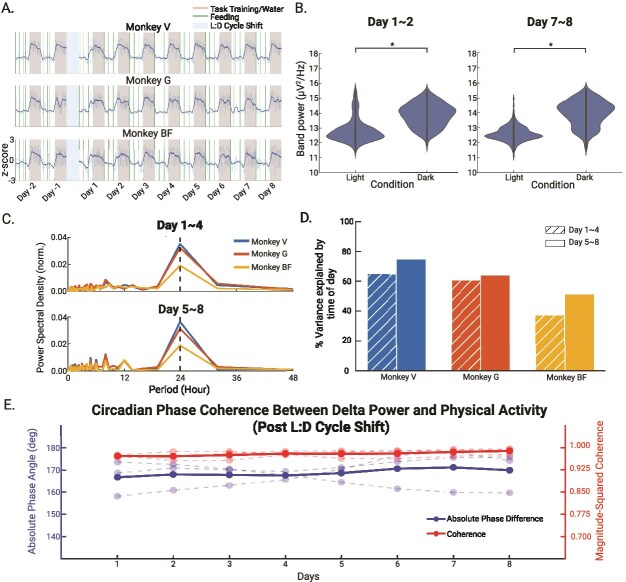
Diurnal patterns of delta power in the shifted condition. (A) Time series of delta power in the shift condition (8 days, *n* = 3). Shaded gray areas indicate dark phases. Blue shaded regions indicate the day of L:D shift. Green and orange lines denote feeding and task training times, respectively. Animals received their water during and right after the task training. We standardized delta power by taking the z-score. (B) Distribution of delta power (1–4 Hz) across light and dark phases on day 1 and 2 and day 7 and 8 post-shift. Asterisks denote significant differences (^*^p < .05). (C) Percentage of variance in delta power explained by time of day (light vs. dark) for each monkey during early (day 1–4) and late (day 5–8) adaptation. (D) Periodograms of delta power time series during early (day 1–4) and late (day 5–8) adaptation. We normalized each trace in the plot by the sum of the power across all periods. The dash vertical line marks the peak power period (24 h) for each monkey. (E) Daily average of magnitude-squared coherence and absolute phase angle between delta power and physician activity post L:D cycle shift (8 days). We computed wavelet coherence between the two signals, extracted values from the 24-h circadian band, and averaged coherence and phase angle for each 24-h bin. Solid lines represent the group mean (*n* = 3); dashed lines show individual animals.

After extending the lights-on for 8 h (shifted condition), we observed a temporary misalignment between the delta power fluctuation and light dark cycle. On the 2 days preceding the L:D cycle shift (day -2 and -1), the rise in delta power was fully synchronized with the light-off time (Figure 3, A). On the 2 days (day 1 and day 2) after the L:D cycle shift, delta power began to rise ~2–3 h before the light-off time (Figure 3, A). The misalignment lasted for 2 days before delta power realigned with the L:D cycle. We did not include the data during the 8-h transition of the L:D cycle shift due to recording pauses required for cage maintenance (see Methods). To quantify changes in delta power dynamics, we performed multiple analyses examining delta power amplitude, rhythmicity, and circadian coupling with physical activity. We found a significant, and consistent increase in delta band power when comparing the dark and light periods immediately after the shift (day 1 and 2: *t*(1718) = 27.75, *p* < .001) and again, after realignment (day 7 and 8: *t*(1725) = 42.10, *p* < .001; Figure 3, B). A significant proportion of the variance in delta power could be explained by time of day fits early after the shift (day 1–4 of the shifted L:D cycle: Monkey V = 65.05%, Monkey G = 60.41%, Monkey BF = 37.41%, *p* < .001, temporal shuffling test). This proportion increased for all three animals after the entrainment period (day 5–8 of the shifted light–dark cycle: Monkey V = 75.09%, Monkey G = 64.33%, Monkey BF = 51.28%, *p* < .001, temporal shuffling test) (Figure 3, C). This increase in variance explained suggests that as animals spent more time in the shifted L:D cycle, their delta power fluctuations became more strongly aligned with the new light cycle, reflecting re-entrainment. More importantly, the dominant period across the shift condition remained consistent at 24-h in day 1–4 and day 5–8 after the light shift (Figure 3, D). This indicates that across the time shifted condition (8 days), the daily rhythm remained intact.

We next examined the phase relationship between delta power and physical activity in the shifted L:D cycle condition using wavelet coherence analysis. The coherence between delta power and physical activity remained consistently high throughout the 8-day recording period (group coherence = 0.98 ± 0.01; individual: Monkey V = 0.98 ± 0.01, Monkey G = 0.98 ± 0.02, Monkey BF = 0.98 ± 0.01; Figure 3, E). The absolute phase difference remained close to 180 degrees (group absolute phase angle = 168.85 ± 1.56; individual: Monkey V = 171.97 ± 2.79, Monkey G = 166.23 ± 5.68, Monkey BF = 168.36 ± 7.57), indicating a robust anti-phase relationship. These results suggest that the coupling between EEG-derived delta power and physical activity is preserved under shifted L:D cycle condition, and that delta power continues to reflect an inverse pattern to behavior.

### *Delta power transition* 

**Figure 4:**
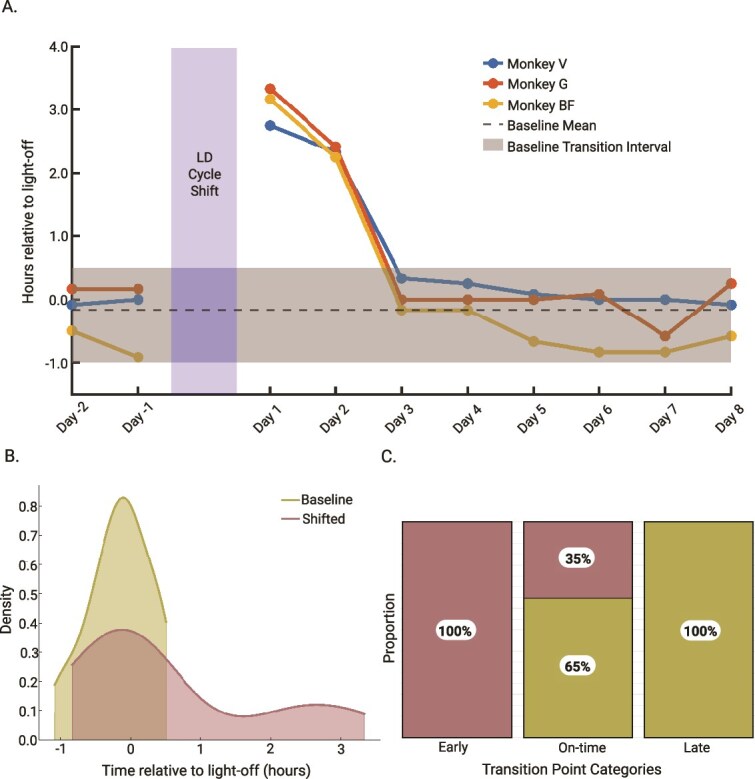
Delta power transition point after a shifted light–dark cycle. (A) Delta Power transition point during the shifted condition. The line plot shows delta power transition points relative to light-off for individual monkeys before and after the shifted light dark cycle. The horizontal dashed line represents the group baseline mean (*n*_baseline_ = 36), and the shaded area represents the range between the 5th percentile and the 95th percentile of baseline Delta power transition point. (B) Distribution of the delta power transition point in baseline (yellow trace) and shift conditions (red trace). The histogram compiles the delta power transition point across all three subjects (*n*_baseline_ = 36, *n*_shift_ = 24). (C) Chi-square test of independence for Delta power transition point categories. The stacked bar plot represents the proportion of delta power transition point *x* categorized as early (*x* > 1), on-time (−1 < *x* < 1), and late (*x* < −1).

After extending the lights-on for 8 h (shifted condition), we also observed that the Delta power transition point shifted earlier by more than 2 h. The Delta power transition point returned to the baseline level 3 days after the light–dark cycle shift (Figure 4, A). The early transition of delta band power in the first 2 days of the shifted condition indicates a temporary misalignment between the slow wave brain activity and the environmental cue (light). However, this misalignment was overcome within 3 days as the animals adapted to the new light–dark cycle. The kernel density plot of delta power transition points shows a clear change in distribution between baseline and shift conditions. Under the baseline condition, transition points are tightly clustered around 0. In the shift condition, the distribution is more spread and skewed to later transition points (Figure 4, B). The distribution of transition point categories shows a significant difference in the distribution of transition point categories (*ꭓ*^2^(2) = 11.47, *p* = .00323, Figure 4, C) with a relatively strong effect (V_Cramér_ = 0.4; 95% CI = [0.00 to 0.65]), with all early transitions (>1 h) coming from the shift condition.

## Discussion

In this study, we implanted three cynomolgus monkeys with telemetry devices. These devices allowed us to continuously monitor the EEG and actigraphy for multiple consecutive days in a stable L:D cycle (lights on: 6:00, lights off: 19:00). We then recorded the same physiological data in a shifted L:D cycle, where we delayed the light-on and light-off by 8 h (light on: 14:00, light off: 3:00). We derived log spectral power in the delta band (0.5–4 Hz) from EEG data and physical activity from actigraphy data. We found that delta power and physical activity exhibited a stable anti-phase relationship (Figures 2, E and 3, E). We found that shifting L:D cycles led to earlier transition of delta power from awake state and rest state, and transition timing realigned with the light-off time as the animals adapted to the new light–dark cycle. This finding highlights the close alignment of slow wave brain activity to environmental cues that govern the sleep–wake cycle (zeitgebers) and the gradual adaptation of such brain rhythm to changes in these zeitgebers.

We found that delta power was consistently lower during the light period compared to the dark period in all three animals (Figures 2, A and B and 3, A and B). This finding reinforces previous observations of diurnal variation in delta power in cynomolgus monkeys derived from single-day EEG recordings [24]. In their study, Rachalski *et al.* found increased delta wave activity as animals transitioned into the dark phase based on continuous 24-h EEG recordings. They concluded that delta power reflects the build-up and dissipation of sleep pressure across the light–dark cycle. Their finding aligns with studies in humans showing that delta activity reflects sleep intensity and homeostatic sleep regulation [26, 38–43]. Extending beyond these earlier observations, our study examined delta power dynamics across several consecutive days and under both baseline and shifted L:D cycle. We found this diurnal pattern to be consistent across days. After the shift of L:D cycle, the difference between light period and dark period remained significant. This indicates that alteration of the L:D cycle does not lead to prolonged changes in the diurnal divergence of delta power in NHPs. Crucially, the magnitude of this diurnal divergence remained consistent between the beginning (day 1 and 2) and the end (day 7 and 8) of the shifted L:D cycle shift. This stability demonstrates that the homeostatic drive remained intact despite the acute shift of light–dark cycle.

To more precisely quantify how tightly delta power remained aligned to time of day during this transition, we analyzed the variance in delta power explained by time-of-day fits across different phases of re-entrainment, an approach not addressed in Rachalski *et al.* We found that even in the first 4 days after the L:D cycle shift, a significant proportion of delta power variability remained linked to the time-of-day (Figure 3, D). Along with the preservation of a 24-h dominant period (Figure 3, C), this finding indicates that the underlying rhythmic structure persisted despite the abrupt environmental change. While the 24-h component was the dominant feature, we also observed secondary peaks around the 8 h range in the periodograms (Figures 2, D and 3, C). These likely reflect harmonics of the fundamental circadian rhythm: because the diurnal delta power signal approximates a square wave, the Fourier transform naturally generates spectral power at odd harmonics of the fundamental frequency. Additionally, we noted inter-subject variability in the stability of the delta power within the light and dark period, especially the fluctuations of delta power observed during the dark phase in Subject G. These likely reflect the intrinsic individual differences in maintaining the resting state. This finding is consistent with the variability in rest-activity patterns previously reported in NHPs [24]. As animals spent more time in the shifted L:D cycle (day 5–8), the variance explained by time-of-day increased further (Figure 3, D). During this same time span, the dominant period of delta power remained at 24 h (Figure 3, C). Together, these findings suggest that delta power gradually re-aligned to the new L:D cycle. This analysis highlights not only the stability of daily rhythmicity but also provides a quantitative measure of the re-entrainment.

We examined the coherence between delta power and physical activity to assess how tightly their oscillations were coupled over a 24-h cycle. Using wavelet coherence analysis, we found consistently high magnitude-squared coherence values across all three animals in both baseline and shifted conditions, indicating strong and stable temporal coupling between delta activity and physical activity. Previous studies have shown circadian coherence between brain activity and peripheral metabolic signals such as interstitial glucose dynamics [44, 45]. Our findings extend this principle by demonstrating strong temporal coupling between neural states and overt behavior across the circadian cycle. Furthermore, the absolute phase difference between the two signals remained near 158° in the baseline and near 170° in the shifted condition, reflecting a robust anti-phase relationship: delta power peaked during rest, while activity peaked during wakefulness. This pattern demonstrates that delta power follows a circadian rhythm that is consistently out of phase with locomotor activity. Importantly, both strength of coherence and the anti-phase relationship between delta power and locomotor activity were preserved following the shift in the light–dark cycle, suggesting that temporal coupling between rest-related brain activity and locomotor behavior is robust to changes in environmental timing cues.

We found a significant change in the timing of delta power activity transition was related to the light–dark cycle and the delayed light-on and light-off time. Specifically, we found an early increase of delta power. In the first 2 days of the shifted light–dark cycle, delta power began to increase before the lights turned off. This early transition to the resting state was 2.5–3.5 h early on the first night and 2.5 h early on the second night. By the third night, we observed that the delta power was once again transitioning to the resting state within 1 h (or min) before lights off. We interpret this as a full adaptation to the new L:D cycle in 3 days. This adaptation was 1–3 days faster than previous reports on humans, rodents, and monkeys subject to an 8 h phase delayed shift [46–48]. One possible explanation for the faster adaptation in our study is that the animals were kept on a strict schedule, providing several zeitgebers to aid in reentrainment. During the baseline conditions, they were fed three times a day at the first hour, fifth–sixth hour, and eighth–ninth hour relative to light on relative to lights on. They were also trained to perform an in-cage training task which was consistently set up 1–3 h after lights on, and each training session lasted for ~1 h. Since they received water as a positive reward for task performance, they also received their daily water intake at consistent times across days. These times, which also acted as environmental cues, also shifted with the new light-on, light-off schedule. Thus, we believe the consistency of additional environmental cues, acting as non-photic zeitgebers and metabolic feedback, may have helped the animals adapt to the new light–dark cycle faster.

There are a few limitations of the current study that prevent us from drawing more comprehensive conclusions about the underlying mechanisms of delta power fluctuations in response to change of light–dark cycle. First of all, we did not utilize a forced desynchronized protocol, a method in which the sleep–wake cycle is systematically misaligned with the circadian clock by controlling the timing of light exposure. This protocol is widely used to separate the effects of circadian clock from other factors such as sleep homeostasis or acute light responses [38]. Therefore, we cannot determine whether the entrainment we observed in the data is solely driven by the intrinsic circadian clock, and thus it is likely that these results reflect a mix of effects from the endogenous circadian clock as well as external stimuli. Additionally, we acknowledge constraints regarding the animal cohort and instrumentation. Our sample size (*n* = 3) is constrained by the ethical considerations and resource limitations in NHP research. In addition, our cohort was exclusively male and could not address the difference in delta power re-entrainment between genders. Future studies should ideally include female subjects to investigate potential sex-based differences in delta power resynchronization to new L:D cycle, as recent evidence in rodents suggests estrogen could accelerate re-entrainment [49]. Furthermore, our EEG recordings were restricted to a few channels per animal, which only provided a local proxy for global cortical synchronization. Given that electrode placement varied between the left and right hemisphere between subjects, we cannot rule out the influence of lateralization on the observed dynamics. Future research should utilize high-density arrays to characterize potential inter-hemispheric or regional differences in the rate of re-entrainment. While the telemetry implants used in this study were capable of monitoring other physiological markers like core body temperature and heart rate, this study focused specifically on cortical delta power. Future multi-modal integration of these biomarkers would provide a broader perspective on interaction of different body clocks during re-entrainment. Finally, our setup did not allow us to take into account how an animal’s temperament or mood may affect the observed biological rhythms. Multiple studies have shown that subjects’ stress and mood could affect the stability of biological rhythms, as well as adaptation to a new light–dark cycle [50–52]. The emotional state of cynomolgus monkeys can be categorized based on their behavioral patterns captured in video [35], but due to limitations in the housing environment, we were unable to record videos that would document the animals’ behavioral patterns. However, despite these limitations, the consistency of delta power fluctuations observed across all animals in both stable and shifted L:D cycle, as well as the consistency in the entrainment of delta power during shifted L:D cycle, strongly suggests that our findings are robust to these potential confounds. The global trend in the data, along with the lack of significant inter-individual variability, supports the conclusion that the observed changes in delta power are driven by a mixed effect of endogenous circadian clock and external environmental cue rather than individual temperament states or other unmeasured factors beyond the scope of our data.

To conclude, we found that delta power fluctuations closely correspond to the animals’ rest-activity patterns, serving as a reliable neural indicator of transitions between wakefulness and sleep. Shifting the L:D cycle induced an earlier transition of delta power from the light phase to the dark phase, reflecting a temporary misalignment of brain activity relative to the new environmental timing cues. Importantly, despite this temporary misalignment, the diurnal divergence in delta power between light and dark periods remained intact, and the dominant 24-h rhythm persisted throughout the shift condition. By quantifying the variance in delta power explained by time of day, we further demonstrated that alignment to the new L:D cycle strengthened over time, providing a quantitative measure of re-entrainment. These findings suggest that while external environmental changes can temporarily influence the timing of brain rhythms related to sleep homeostasis, the underlying circadian organization remains robust and adaptable. This work offers new insight into how neural and behavioral states in NHPs adjust flexibly to environmental disruptions while maintaining core rhythmic stability.

## Data Availability

Data reported in this paper will be shared upon request.
